# Enhanced Power Conversion Efficiency of Perovskite Solar Cells with an Up-Conversion Material of Er^3+^-Yb^3+^-Li^+^ Tri-doped TiO_2_

**DOI:** 10.1186/s11671-018-2545-y

**Published:** 2018-05-11

**Authors:** Zhenlong Zhang, Jianqiang Qin, Wenjia Shi, Yanyan Liu, Yan Zhang, Yuefeng Liu, Huiping Gao, Yanli Mao

**Affiliations:** 1 School of Physics and Electronics, Henan University, Kaifeng, 475004 China; 2 Institute of Micro/Nano Photonic Materials and Applications, Henan University, Kaifeng, 475004 China

**Keywords:** Enhanced power conversion efficiency, Perovskite solar cells, Up-conversion material

## Abstract

In this paper, Er^3+^-Yb^3+^-Li^+^ tri-doped TiO_2_ (UC-TiO_2_) was prepared by an addition of Li^+^ to Er^3+^-Yb^3+^ co-doped TiO_2_. The UC-TiO_2_ presented an enhanced up-conversion emission compared with Er^3+^-Yb^3+^ co-doped TiO_2_. The UC-TiO_2_ was applied to the perovskite solar cells. The power conversion efficiency (PCE) of the solar cells without UC-TiO_2_ was 14.0%, while the PCE of the solar cells with UC-TiO_2_ was increased to 16.5%, which presented an increase of 19%. The results suggested that UC-TiO_2_ is an effective up-conversion material. And this study provided a route to expand the spectral absorption of perovskite solar cells from visible light to near-infrared using up-conversion materials.

**Electronic supplementary material:**

The online version of this article (10.1186/s11671-018-2545-y) contains supplementary material, which is available to authorized users.

## Background

Organolead halide perovskite solar cells (PSCs) have become attractive in the solar cell field, which is due to their advantages, such as high efficiency, lost cost, and simple fabrication [1–4]. In a few years, the power conversion efficiency (PCE) of PSCs has been improved to 22.1% [5]. However, perovskite solar cells only absorb a small fraction of incident light in UV and visible ranges due to the narrow energy band gap of perovskite sensitizer; thus, a large portion of incident light is lost due to its non-absorption of near-infrared (NIR) [6, 7].

One promising method to solve the NIR energy loss issue is to apply up-conversion materials to PSCs, which can convert NIR to visible light. Some authors have reported the applications of up-conversion materials to perovskite solar cells [8–10], in which the up-conversion materials adopted were mainly based on beta-phase sodium yttrium fluoride (β-NaYF_4_). While the β-NaYF_4_ up-conversion materials can reduce charge transport ability of electron transfer layer [11]. It has been reported that Er^3+^-Yb^3+^-F^−^ tri-doped TiO_2_ can improve the PCE of dye-sensitized solar cells (DSSCs) due to its enhanced up-conversion emission compared with Er^3+^-Yb^3+^ co-doped TiO_2_ [12]. In our previous publication [13], we reported the application of Er^3+^-Yb^3+^ co-doped TiO_2_ nanorods to PSCs. Some researchers have proved that the addition of Li^+^ into Er^3+^-Yb^3+^ co-doped TiO_2_ could increase the up-conversion emission [14, 15]. And it has been reported that the perovskite solar cells based on Li-doped TiO_2_ produce higher performances compared to the device based on un-doped TiO_2_ [16]. Thus, we wonder if the up-conversion materials of Er^3+^-Yb^3+^-Li^+^ tri-doped TiO_2_ can be applied to PSCs to further improve the performance.

Therefore, in the present study, we prepared Er^3+^-Yb^3+^-Li^+^ tri-doped TiO_2_ (UC-TiO_2_) by addition of Li^+^ into Er^3+^-Yb^3+^ co-doped TiO_2_, which presented an enhanced up-conversion emission compared with Er^3+^-Yb^3+^ co-doped TiO_2_. The UC-TiO_2_ was applied to perovskite solar cells. The PCE of the solar cells with UC-TiO_2_ is increased to 16.5 from 14.0% for the solar cells without UC-TiO_2_, which presents an increase of 19%.

## Methods

### Synthesis of Er^3+^-Yb^3+^-Li^+^ Tri-Doped TiO_2_

The nanocrystals of Er^3+^-Yb^3+^-Li^+^ tri-doped TiO_2_ (UC-TiO_2_) were synthesized by a modified method [15]. A titanium (IV) n-butoxide was prepared by mixing n-butyl titanate (Ti(OBu)_4_) with acetylacetone (AcAc) at room temperature for 1 h under agitation. Then, the iso-propyl (i-PrOH) was put in the titanium (IV) n-butoxide. Next, i-PrOH, deionized water, and concentrated nitric acid (HNO_3_) was mixed and dropped into the solution. A light yellow TiO_2_ sol was obtained after stirring for 6 h. The molar ratios of AcAc, H_2_O, and HNO_3_ to Ti(OBu)_4_ were 1:1, 2:1, and 0.3:1, respectively. Then, Er(NO_3_)_3_·5H_2_O, Yb(NO_3_)_3_·5H_2_O, and LiNO_3_ were added into the TiO_2_ sol to make the molar ratios of Er:Yb:Li:Ti = 0.5:10:*x*:100 (*x* = 0, 10, 15, 20, 25). The solvent in the Er^3+^-Yb^3+^-Li^+^ tri-doped TiO_2_ sol (UC-TiO_2_ sol) was removed by drying for 8 h at 100 °C. Then, the UC-TiO_2_ was calcined at 500 °C for 30 min.

### Fabrication of Perovskite Solar Cells

Patterned FTO glass substrate was cleaned in acetone, 2-propanol, and ethanol by sonication for 20 min, respectively. Then, UV-O_3_ was used to treat the FTO for 15 min. A compact layer was formed by spin-coating a precursor solution on FTO and annealed at 500 °C for 30 min. The precursor solution is 0.1 M titanium diisopropoxide bis (acetylacetonate) (75 wt% in isopropanol, Aldrich) solution in 1-butanol. A mesoporous TiO_2_ film was obtained by spin-coating TiO_2_ solution on the compact layer at 4000 rpm for 30 s, followed by annealing at 100 °C for 10 min and 500 °C for 30 min, respectively. The TiO_2_ solution was prepared by diluting TiO_2_ paste (30NR-D, Dyesol) with ethanol (1:6, weight ratio) or by mixing the UC-TiO_2_ sol and the diluted TiO_2_ solution (UC-TiO_2_:TiO_2_ = *x*:100, *v*/*v*, *x* = 10, 20, 30, and 40). A perovskite layer was formed on UC-TiO_2_ layer by spin-coating perovskite precursor solution in two steps at 1000 rpm for 10 s and 4000 rpm for 30 s, and 200 μL chlorobenzene was poured on the substrate during the second step before the end of 20 s. Then, the sample was heated on the hotplate at 100 °C for 1 h. The perovskite precursor solution was obtained by mixing PbI_2_ (1.1 M), FAI (1 M), PbBr_2_ (0.22 M), and MABr (0.2 M) in the mixed solvent of anhydrous DMF/DMSO (4:1 *v*:*v*). Then, a 1.5 M stock solution of CsI pre-dissolved in DMSO was dropped into the mixed perovskite precursor solution [17]. A hole-transfer layer was formed by spin-coating a solution of spiro-MeOTAD at 4000 rpm 30 s. Finally, an 80-nm-thick gold layer was thermally evaporated on the top of the device.

### Characterization

Up-conversion fluorescence, steady-state photoluminescence (PL), and time-resolved photoluminescence (TRPL) spectra were measured with a FLS 980 E fluorometer (Edinburgh Photonics). X-ray diffraction (XRD) spectra were acquired on a diffractometer (DX-2700). X-ray photoelectron spectroscopy (XPS THS-103) with Al Ka as X-ray source was applied to measure the chemical state of the samples. Ultraviolet visible near-infrared (UV-vis-NIR) absorption spectra were collected on a Varian Cary 5000 spectrophotometer. Microstructure and morphologies were observed with a scanning electron microscope (SEM; JEM-7001F, JEOL). Photocurrent-voltage (J-V) curves were measured with a Keithley 2440 Sourcemeter under AM 1.5 G illumination. The electrochemical impedance spectroscopy (EIS) was obtained with an electrochemical workstation (CHI660e, Shanghai CHI Co., Ltd.).

## Results and Discussion

**Fig. 1:**
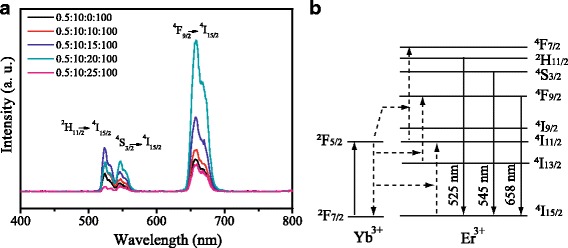
**a** Up-conversion emission spectra of Er^3+^-Yb^3+^-Li^+^ tri-doped TiO_2_ (Er:Yb:Li:Ti = 0.5:10:*x*:100, *x* = 0, 10, 15, 20, and 25, molar ratio). **b** Schematic energy-transfer mechanisms of the Er^3+^ ions

Up-conversion emissions were measured with an excitation of a 980-nm laser. Figure 1a shows the up-conversion emissions spectra of Er^3+^-Yb^3+^-Li^+^ tri-doped TiO_2_ (Er:Yb:Li:Ti = 0.5:10:*x*:100, *x* = 0, 10, 15, 20, and 25, molar ratio). Figure 1b shows the schematic energy-transfer mechanisms of the Er^3+^ ions. The green emission bands located at about 525 and 545 nm can be attributed to ^2^H_11/2_→^4^I_15/2_ and ^4^S_3/2_→^4^I_15/2_ transitions of Er^3+^ ions, respectively. And the red emission bands centered at about 658 nm could correspond to ^4^F_9/2_→^4^I_15/2_ transitions of Er^3+^ ions [15, 16]. With the increase of Li^+^ doping content, the intensity of the spectrum increases firstly, and then decreases, which is the maximum when the doping content of Li^+^ is *x* = 20. Hereinafter, the up-conversion material of Er^3+^-Yb^3+^-Li^+^ tri-doped TiO_2_ (Er:Yb:Li:Ti = 0.5:10:20:100, molar ratio) was applied.

**Fig. 2:**
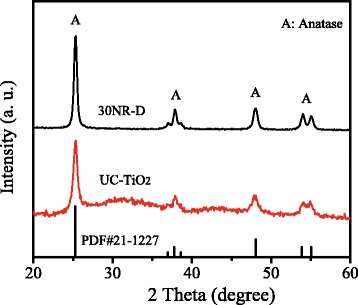
XRD patterns of commercial TiO_2_ (30NR-D) and UC-TiO_2_

Figure 2 displays the XRD patterns of commercial TiO_2_ (30NR-D) and UC-TiO_2_. The XRD pattern of UC-TiO_2_ is similar to that of 30NR-D. The peaks at 25.3°, 37.8°, 48.0°, and 53.8° in the XRD patterns are assigned to the (101), (004), (200), and (105) planes (JCPDS card no.21-1272), respectively, which indicates that UC-TiO_2_ and 30NR-D belong to the anatase phase of TiO_2_.

**Fig. 3:**
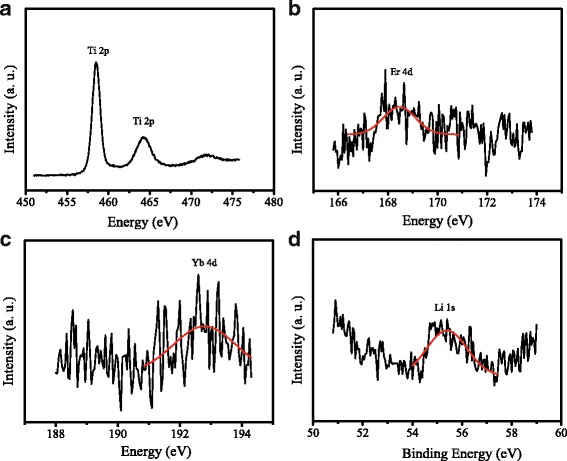
XPS spectra of UC-TiO_2_. **a** Ti 2p, **b** Er 4d, **c** Yb 4d, and **d** Li 1s

To confirm the doping of Er, Yb, and Li into TiO_2_, XPS spectra of UC-TiO_2_ were recorded and shown in Fig. 3. The peaks at 458.1 and 463.9 eV in Fig. 3a could belong to Ti 2p_3/2_ and Ti 2p_1/2_, respectively, and the peaks at 168.8 eV in Fig. 3b and at 192.7 eV in Fig. 3c could be attributed to Er 4d and Yb 4d, respectively [18]. The peak at 55.5 eV in Fig. 3d can correspond to Li 1s [19]. The survey XPS spectrum of UC-TiO_2_ and O1s peak were also presented in Additional file 1: Figure S1. The results demonstrated that Er, Yb, and Li atoms were doped into TiO_2_.

**Fig. 4:**
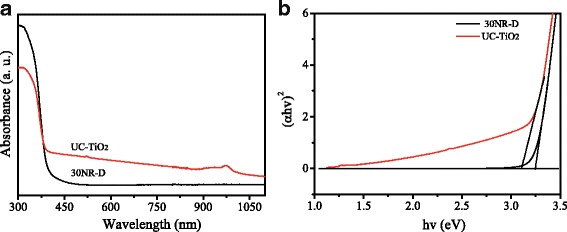
**a** UV-vis-NIR absorption spectra of commercial TiO_2_ (30NR-D) and UC-TiO_2_. **b** Tauc plots

Figure 4a displays the UV-vis-NIR absorption spectra of TiO_2_ (30NR-D) and UC-TiO_2_. Compared with 30NR-D, UC-TiO_2_ presents a stronger absorption, especially at the range from 900 to 1000 nm. The energy band gap (*E*_g_) could be estimated with the Tauc plot [20]. The Tauc plots are shown in Fig. 4b, from which the values of *E*_g_ could be obtained to be 3.20 and 3.10 eV for 30NR-D and UC-TiO_2_, respectively. The *E*_g_ of UC-TiO_2_ is smaller than that of un-doped TiO_2_.

**Fig. 5:**
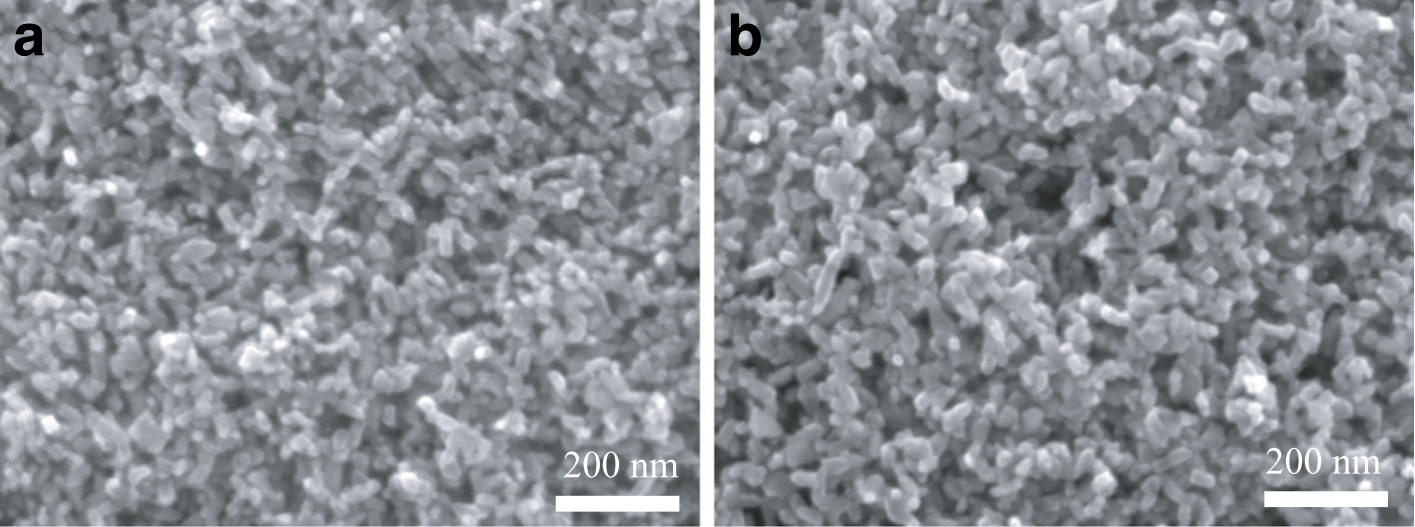
SEM images of the mesoporous layers. **a** 30NR-D film without UC-TiO_2_. **b** 30NR-D film with UC-TiO_2_

Figure 5a shows the SEM image of 30NR-D film formed on the compact layer. The nanoparticle size is about 30 nm, and the size distribution is uniform. Figure 5b shows the SEM image of the film containing 30NR-D and UC-TiO_2_ deposited on the compact layer by spin-coating method. There is no obvious difference between the two films, which displays that the particle size and morphology of UC-TiO_2_ are similar to those of 30NR-D.

In the present work, the perovskite film was formed with the method previously reported [17]. According to report, the composition of the perovskite film is Cs_5_(MA_0.17_FA_0.83_)_95_Pb(I_0.83_Br_0.17_)_3_, and the role of the CsI is to make the perovskite solar cells thermally more stable, with less phase impurities, and less sensitive to processing conditions [17]. The scheme of the device is presented in Additional file 1: Figure S2.

**Fig. 6:**
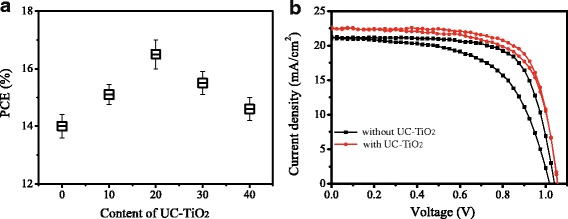
**a** PCE dependence of solar cells on the content of UC-TiO_2_ (UC-TiO_2_: 30NR-D = *x*:100, *v*/*v*) in the mixture. **b** I-V curves of the best performance devices without UC-TiO_2_ and with UC-TiO_2_

The perovskite solar cells based on mesoporous layer formed with the mixture of UC-TiO_2_ sol and diluted TiO_2_ solution (UC-TiO_2_:TiO_2_ = *x*:100, *v*/*v*, *x* = 0, 10, 20, 30, and 40) were fabricated and their I-V curves were measured. The photovoltaic parameters were obtained from the I-V measurements. Figure 6a shows the PCE dependence of solar cells on the content of UC-TiO_2_ (UC-TiO_2_:TiO_2_ = *x*:100, *v*/*v*) in the mixture. With the increase of UC-TiO_2_ content, the power conversion efficiency (PCE) of solar cells increases firstly, and then decreases, which is the maximum at the content of *x* = 20 for UC-TiO_2_. The detailed photovoltaic parameters of solar cells with 20% UC-TiO_2_ and without UC-TiO_2_ were listed in Table 1. Compared with those of the devices without UC-TiO_2_, the photovoltaic parameters of the solar cells with UC-TiO_2_ present an improvement. The PCE of the solar cells with 20% UC-TiO_2_ is increased to 16.5 from 14.0% for the solar cells without UC-TiO_2_, which presents an increase of 19%. Figure 6b displays the I-V curves of the typical solar cells with UC-TiO_2_ and without UC-TiO_2_.

**Table 1:** Photovoltaic parameters of the solar cells with UC-TiO_2_ (20%) and without UC-TiO_2_

Samples	*V*_oc_ (*V*)	*I*_sc_ (mA/cm^2^)	FF (%)	PCE (%)
Control	1.01 ± 0.01	21.0 ± 0.2	66.0 ± 0.3	14.0 ± 0.3
With UC-TiO_2_	1.05 ± 0.01	22.2 ± 0.3	70.8 ± 0.02	16.5 ± 0.2

**Fig. 7:**
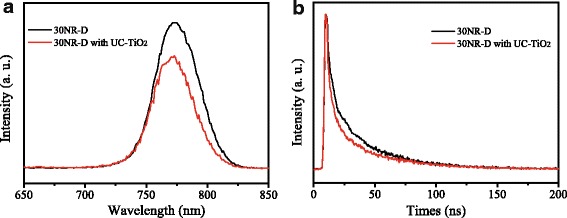
**a** PL and **b** TRPL of perovskite layers on the mesoporous layers formed by 30NR-D without UC-TiO_2_ and 30NR-D with UC-TiO_2_

To understand the enhancement, some investigations were carried out. Steady-state photoluminescence (PL) and time-resolved photoluminescence (TRPL) can be applied to investigate the electron extration and transport process. The PL of perovskite layer on the mesoporous layers formed by 30NR-D and 30NR-D with UC-TiO_2_ were measured and shown in Fig. 7a. Compared with that of 30NR-D/perovskite, the PL intensity of 30NR-D with UC-TiO_2_/perovskite becomes reduced, which indicates that the electron extration and transport efficiency across the interface between 30NR-D with UC-TiO_2_ and perovskite is better than that between 30NR-D and perovskite [21]. Figure 7b shows the TRPL spectra of perovskite layer on the mesoporous layers formed by 30NR-D and 30NR-D with UC-TiO_2_. The TRPL spectrum was fitted to a biexponential function, in which the fast decay (τ_1_) may be resulted from transportation of free carriers, and the slow decay (τ_2_) can be originated from radiative recombination of free carriers [22–24]. The obtained parameters are listed in Table 2. Compared with that of 30NR-D/perovskite, the fast decay time (2.8 ns) of 30NR-D with UC-TiO_2_/perovskite becomes smaller, while the fraction of fast decay process (98.2%) becomes larger. This implies that the charge transfer between perovskite and 30NR-D with UC-TiO_2_ is faster than that between perovskite and 30NR-D.

**Table 2:** Parameters of the TRPL spectra

Samples	τ_1_/ns	% of τ_1_	τ_2_/ns	% of τ_2_
30NR-D/perovskite	3.6	94	31.7	6.0
30NR-D with UC-TiO_2_/perovskite	2.8	98.2	31.0	1.8

**Fig. 8:**
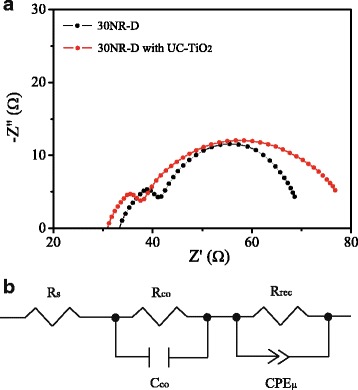
**a** Nyquist plots of the devices based on mesoporous layers formed by 30NR-D without UC-TiO_2_ and 30NR-D with UC-TiO_2_. **b** Equivalent circuit applied to fit the EIS data

Eletrochemical impedance spectroscopy (EIS) is an effective method to get some information on carrier transfer behavior. Figure 8a displays the Nyquist plots of the devices based on mesoporous layers formed by 30NR-D and 30NR-D with UC-TiO_2_, in which two arcs were observed. The arc at high-frequency could be resulted from the contact resistance between the interfaces, and the arc at low-frequency could come from the recombination resistance (*R*_rec_) and chemical capacitance (*C*_μ_) of the device [25, 26]. The EIS was fitted with an equivalent circuit shown in Fig. 8b, and the obtained parameters are listed in Table 3. The series resistance of the devices based on 30NR-D with UC-TiO_2_ becomes smaller than that of the devices on based on 30NR-D, while the recombination resistance of the former becomes larger than that of the later. This indicates the charge recombination was decreased and the charge transport was improved for the device based on 30NR-D with UC-TiO_2_.

**Table 3:** Fitting parameters for EIS data

Solar cells	*R*_s_/Ω	*R*_co_/Ω	*R*_rec_/Ω	*C*_co_/Ω	CPE-T/F	CPE_μ_-P
30NR-D	34.1	10.3	31.2	1.5E-7	5.9E-6	0.80
30NR-D with UC-TiO_2_	31.5	9.3	46.8	1.8E-7	5.5E-5	0.61

To further prove the effect of UC-TiO_2_ on photocurrents of the devices, the I-V curves of the devices based on the mesoporous layers without UC-TiO_2_ and with UC-TiO_2_ were measured under the simulated solar radiation in the wavelength range of *λ* ≥ 980 nm with a NIR filter, which are shown in Additional file 1: Figure S3. Compared with that of the device without UC-TiO_2_, the photocurrent of the device with UC-TiO_2_ was obviously enhanced, which demonstrates that the incorporation of UC-TiO_2_ in the device can transform the NIR light into visible light, which can be absorbed by the devices to generate photocurrent.

**Fig. 9:**
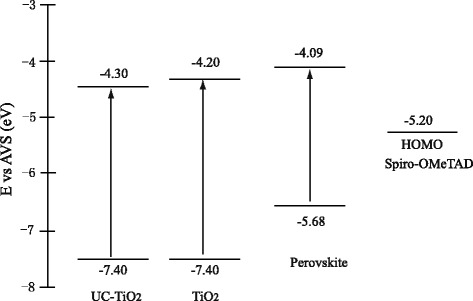
Schematic energy band arrangements of UC-TiO_2_, TiO_2_, perovskite, and Spiro-OMeTAD

To explain the increased open circuit voltage (*V*_oc_) of the solar cells, the energy band arrangements of UC-TiO_2_, TiO_2_, perovskite, and Spiro-OMeTAD were shown in Fig. 9 based on the absorption spectra (Fig. 4) and the literatures [27, 28]. The conduction band edge of the UC-TiO_2_ is lower than that of TiO_2_ (30NR-D) due to its smaller energy band gap; thus, the conduction band offset between UC-TiO_2_ and perovskite is larger than that between TiO_2_ and perovskite. This could be one of the reasons to have a higher open circuit voltage for UC-TiO_2_ based solar cells [29, 30].

In summary, the PCE increase of the solar cells based on the mesoporous layer with UC-TiO_2_ is due to the enlarged *I*_sc_ and increased *V*_oc_. The enlarged *I*_sc_ could be due to expansion of spectral absorption to near-infrared (NIR) range with up-conversion material, reduced recombination, and fast charge transfer of photo-generated carriers. The increased *V*_oc_ may be attributed to the enlarged conduction band offset between UC-TiO_2_ and perovskite.

## Conclusions

Er^3+^-Yb^3+^-Li^+^ tri-doped TiO_2_ (UC-TiO_2_) was prepared by addition of Li^+^ into Er^3+^-Yb^3+^ co-doped TiO_2_, which presented an enhanced up-conversion emission. The UC-TiO_2_ was applied to the perovskite solar cells. The performance of solar cells with UC-TiO_2_ was improved compared with that of control device. The *I*_sc_, *V*_oc_, and FF of solar cells with UC-TiO_2_ were increased to 22.2 mA/cm^2^, 1.05 V, and 70.8% from 21.0 mA/cm^2^, 1.01 V, and 66.0% for the control devices, respectively. Thus, the PCE with UC-TiO_2_ was increased to 16.5 from 14.0% for the solar cells without UC-TiO_2_, which presents an increase of 19%. Based on some experimental results, this PCE increase was explained.

## Additional file

Additional file 1: **Figure S1.** a Survey XPS spectrum of UC-TiO2. b O 1s peak. Figure S2 Scheme of the perovskite solar cells. Figure S3 I-V curves of the devices based on the mesoporous layers without UC-TiO_2_ and with UC-TiO_2_ measured under the simulated solar radiation in the wavelength range of *λ* ≥ 980 nm with a NIR filter. (DOCX 81 kb)

## Abbreviations

EIS: Electrochemical impedance spectroscopy
NIR: Near-infrared
PCE: Power conversion efficiency
PL: Photoluminescence
PSCs: Perovskite solar cells
TRPL: Time-resolved photoluminescence
