# Supplementary material for: Enhanced Power Conversion Efficiency of Perovskite Solar Cells with an Up-Conversion Material of Er3+-Yb3+-Li+ Tri-doped TiO2
Source: Nanoscale Res Lett. 2018 May 11;13:147. doi: 10.1186/s11671-018-2545-y (PMC5948189; doi:10.1186/s11671-018-2545-y)
Supplement: Supplementary file 1 — Figure S1. a Survey XPS spectrum of UC-TiO2. b O 1s peak. Figure S2 Scheme of the perovskite solar cells. Figure S3 I-V curves of the devices based on the mesoporous layers without UC-TiO2 and with UC-TiO2 measured under the simulated solar radiation in the wavelength range of λ ≥ 980 nm with a NIR filter. (DOCX 81 kb) [file 11671_2018_2545_MOESM1_ESM.docx]

**Supporting Information**

**Enhanced Power Conversion Efficiency of Perovskite Solar Cells with an Up-conversion Material of Er^3+^-Yb^3+^-Li^+^ Tri-doped TiO_2_**

Zhenlong Zhang ^a^, Jianqiang Qin ^a^, Wenjia Shi ^a^, Yanyan Liu ^a^, Yan Zhang ^a^, Yuefeng Liu ^a^, Huiping Gao ^a^, Wenjia Shi ^a^, Yanli Mao ^a,b,*^

^a^ School of Physics and Electronics, Henan University, Kaifeng 475004, China

^b^ Institute of Micro/Nano Photonic Materials and Applications, Henan University

Figure S1 (a) Survey XPS spectrum of UC-TiO2. (b) O 1s peak.


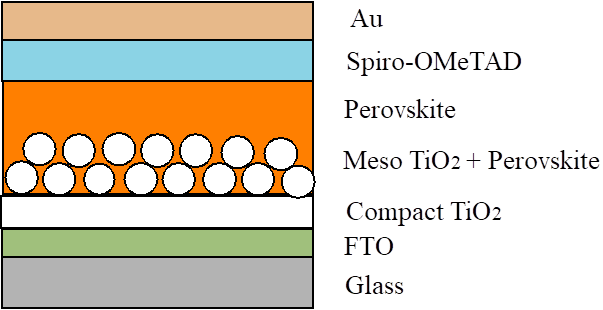


Figure S2 Scheme of the perovskite solar cells.

Figure S3 I-V curves of the devices based on the mesoporous layers without UC-TiO_2_ and with UC-TiO_2_ measured under the simulated solar radiation in the wavelength range of λ≥980 nm with a NIR filter.
